# Cell Lineage Choice during Haematopoiesis: In Honour of Professor Antonius Rolink

**DOI:** 10.3390/ijms19092798

**Published:** 2018-09-17

**Authors:** Geoffrey Brown, Rhodri Ceredig

**Affiliations:** 1 Institute of Clinical Sciences, Institute of Immunology and Immunotherapy, College of Medical and Dental Sciences, University of Birmingham, Edgbaston, Birmingham B15 2TT, UK; 2 Discipline of Physiology, College of Medicine & Nursing Health Science, National University of Ireland, Galway H91 TK33, Ireland; rhodri.ceredig@nuigalway.ie

This volume of the *International Journal of Molecular Sciences* contains a collection of articles by colleagues of Antonius (Ton) Gerardus Rolink (19 April 1953–6 August 2017). Ton had participated in an FP7 Marie Curie Initial Training Network called DECIDE (decision-making within cells and differentiation entity therapies), and the DECIDE partners have submitted articles for this Special Issue. Articles have also been submitted by scientists outside the DECIDE network. We would like to thank all authors for their valuable contributions to this volume.

The DECIDE (decision-making within cells and differentiation entity therapies) network initially arose from the shared interests of Drs Ceredig, Brown, and Rolink in the subject of the process of blood cell formation, or haematopoiesis, and in particular, how progenitor cells differentiate to give rise to the different blood cell types. Haematopoiesis is an archetype cell-lineage system with which to study cell-lineage choice. Research in this area is driven by the need to understand this process at the basic scientific level, as well as abnormal haematopoiesis, in particular, the development of leukaemia. Based largely upon the sequential appearance of blood cells during ontogeny and the close and pairwise relationships between the blood cell types, Brown had initially proposed a radically different (developmental) view of haematopoiesis [1]. In addition, work with cell lines had demonstrated that the B-cell and myeloid lineages were more closely related than what was envisaged by the “classical” model of haematopoiesis. In this model, after an early bifurcation, lymphocytes derive from a common lymphocyte progenitor (CLP), whereas myeloid cells derive from a common myeloid progenitor (CMP). Clearly, cloned cell lines with both lymphoid and myeloid potential posed a problem for the classical model. Subsequently, the work by Amanda Fisher from Ceredig’s laboratory [2] showed that cloned lymphoid tumours arising in an interleukin-7 (IL-7) transgenic mouse line possessed both lymphoid and myeloid potentials. Finally, the seminal papers from Ton’s laboratory describing cell lines deficient in the transcription factor Pax-5 and with a multi-lineage (including lymphoid, in this case T-cell and myeloid) potential also contributed to the need to rethink models of haematopoiesis [3,4].

Thus, after some initial discussions, we three (Ceredig, Rolink, and Brown) decided to put some ideas into writing, and in 2009, we published an opinion article in Nature Reviews Immunology [5]. Based on ideas expressed in this article, we then decided to apply for funding in order to address these ideas. After several iterations and the inclusion of chemists and pharmacists interested in using vitamin D analogues for the control of myeloid cell differentiation and the treatment of leukaemia, funding for the Marie Curie DECIDE network was finally approved in 2013.

In all of the above processes, Ton showed immense enthusiasm for the science. In the meantime, his laboratory had identified an apparently homogeneous bone marrow-derived progenitor population with both lymphoid and myeloid potential, and termed it early progenitor with lymphoid and myeloid potential, or EPLM. With the help of two early stage researchers employed by the DECIDE network working in Ton’s laboratory (Audrey Lilly von Münchow and Llucia Alberti Servera) and two in Brown’s group (Ciaran Mooney and Alan Cunningham), it now transpires that the original EPLM population contains four phenotypically distinct subpopulations. At the genotypic level, the earliest EPLM subpopulation contains individual cells already committed to either lymphoid or myeloid lineage, with essentially no bipotent cells [6].

It must be mentioned that Ton made a massive contribution to the DECIDE network. His enthusiasm for the science has already been mentioned, and this continued throughout the lifetime of DECIDE, even when ill health prevented his full participation. Ton was always prepared to give advice to other members of the DECIDE consortium on projects that were not necessarily aligned with his own research interests. This typifies Ton’s immense generosity and profound knowledge of areas of science outside his immediate domain. His participation in and organization of DECIDE meetings was much appreciated (Figure 1). All 12 Marie Curie Fellows were in receipt of his encouragement and advice regarding their projects. Unfortunately, from early 2017, health issues prevented his participation in the last DECIDE meeting held in Galway.

All of the DECIDE partners are eternally grateful for having had the privilege of knowing and working with Ton, and we dedicate this Special Issue to his memory. In this issue, five DECIDE partners—Brown, Ceredig, Kutner, Sánchez-García, and Marcinkowska—describe some of their findings from the DECIDE work. In 2009, Brown, Ceredig, and Rolink first published their pairwise model for haematopoiesis, and this formed the major scientific basis for establishing the DECIDE consortium [6]. The pairwise model replaces the “classical”, bifurcating lineage tree models with a continuum-like view of the spectrum of fate options open to each hematopoietic stem cell (HSC). In tree models, the progeny of HSCs progress through a series of intermediate hematopoietic progenitors, progressively closing down the lineage options. In the pairwise model, each HSC either self-renews or chooses directly from all of the end-cell options, and then “merely” differentiates. HSCs are also versatile; even soon after their progeny has selected a lineage, they can still step “sideways” to adopt alternative, closely related fates. In their review article in this issue, Brown and Ceredig examine the importance of the developmental ancestry and environmental nurture of HSCs, and argue that stem and progenitor cells are sensitive to lineage guidance by environmental cues. Thus, a cell’s environmental history is important to the specification of lineage [7].

A proposition by Sánchez-García, developed from studies using in vivo models of leukaemogenesis and of carcinomas, relates to the role(s) of oncogenes in the leukaemia and cancer cells-of-origin. He argues that some oncogenes prime the epigenome of leukaemia-initiating cells, but they need not be active thereafter for tumour progression and maintenance. This oncogene “hit” programs the HSC epigenome towards a defined leukemic cell fate. All of the progeny of the resulting clonal leukaemia stem cells (LSCs) then progress towards one leukemic lineage. Vicente-Dueñas, Sánchez-García, and colleagues review the notion of epigenetic stem cell rewiring as a driver of cancer, emphasising that this mechanism represents a common mechanism at work in epithelial tumours [8]. In the case of leukaemia, rewiring fixes the identity of leukaemia cells at the level of HSCs. As HSCs are versatile, an interesting proposition is that the acquisition of a stable oncogene-initiated block to such lineage versatility is a key initiating step to the generation of LSCs and cancer stem cells. This may be a cardinal feature of cancer.

1α,25-dihydroxyvitamin D_3_ (1,25D3)—a physiologically active metabolite of vitamin D—is a potent differentiating agent for both normal and malignant cells, and vitamin D prevents malignant transformation and reduces tumour progression in experimental models, and may be important in human disease. 1,25D3 is also a central regulator of calcium homeostasis, with high doses leading to hypercalcaemia. During DECIDE, Kutner designed and synthesised a panel of novel analogues of vitamin D2, which separate the differentiating and calcaemic actions of 1,25D3, and are substantially more potent differentiating agents. At the DECIDE meetings, Ton often asked the question of how could this be, with just the one receptor for 1,25D3, namely the vitamin D receptor (VDR)? Presently, we do not know the answer to this important question. Kutner and Brown outline some of the rules for eliminating calcaemic action while retaining potency for cell differentiation. A-ring chair β-conformation and (24*E*) side geometry are important for differentiating activity, an aromatic modification of the CD-ring reduces the calcaemic action, and a rigid and straight (24*E*) sidechain confers resistance to catabolism [9].

1,25D3 and all-trans-retinoic acid (ATRA), the active metabolite of vitamin A, are both inducers of myeloid differentiation. Marcinkowska and colleagues have shown that the retinoic acid receptor α (RAR α, for ATRA) regulates VDR expression, and that the outcome of this interaction depends on the developmental status of the cells. For KG-1 (stem-like) and NB-4 (pro-myeloid) cells, activated RAR α upregulates the VDR expression, rendering the KG-1 cells sensitive to 1,25D3-driven differentiation towards monocytes. HL60 and U937 cells typify later myeloid development and, by contrast, an activated RAR α down-regulates VDR [10]. The CCAAT/enhancer-binding protein (C/EBP) family of transcription factors is important for the expression of myeloid-associated genes. Marchwicka and Marcinkowska report that ATRA induces *CEBPB* and *CEBPE* expression; a high level of RAR α results in a strong and sustained *CEBPB* expression. A high VDR expression is required for the strong and sustained upregulation of the *CEBPB* gene, whereas a moderate level of active VDR is sufficient for the expression of *CEBPD*. *CEBPB* is, therefore, the major VDR- and RAR α-regulated gene among the C/EBP family [11]. The transcription factor GATA-1 is important for the erythroid differentiation, which also requires an adequate supply of iron for haemoglobin production. The ferritin heavy subunit maintains iron in a non-toxic form. The article by Zolea and colleagues reveals that this protein does not merely act as a mere iron-metabolism-related factor. Instead, in response to the inducer haemin and via the miR-150 up-regulation and repression of GATA-1, the silencing of the ferritin heavy subunit in the K562 erythroid/myeloid cells blocks the commitment of these cells to erythroid differentiation [12].

For many years, Ton worked on B-cell development, providing important information with which to unravel this process. The review by Sigvardsson brings to attention the developmental trajectories to B-cell development, the complex regulatory networks, and the targeting of the networks in human B-lineage malignancies [13]. Nature versus nurture considerations highlight the roles of bone marrow niches in the development of B-cells from HSCs. Thus, the article by Aurrand-Lions and Mancini emphasises the importance of the marrow environment in maintaining stem cells, as well as their differentiation into mature cells. Cross talk between B-cells and the niches for early pro-B, pre-B, immature B, recirculating B, and plasma cells, either via direct contact and/or secreted specific factors, all contribute to a dynamic process, which is important for the commitment and differentiation of hematopoietic stem and progenitor cells towards a particular pathway [14]. Interleukin-7 (IL-7) is essential for B- and T-lymphocyte development, although there appears to be a species difference in the dependence of B lymphopoiesis. The article by Kasai and colleagues identifies a cytoplasmic region of the mouse IL-7 receptor α subunit (IL-7R α) that is essential for B-cell development, as revealed by a series of deletion mutants of IL-7R α. Amino acids 414–441 in the IL-7Rα chain form a critical subdomain [15]. Studies of antibody-secreting plasma cells have been continuously hampered by the lack of surface molecules with which to identify them. The article by Trezise and colleagues reports mining of the transcriptome of plasma cells to identify novel and cell surface proteins. Three surface proteins, Plpp5, Clptm1l, and Itm2c, represent potential targets for novel treatments for multiple myeloma, a tumour of antibody-secreting cells. In this regard, and as revealed by the analysis of mouse strains with a loss-of-function mutation for each protein, these proteins are dispensable for normal B-cell development and antibody production by antibody secreting cells [16]. Lastly, the review article by Urbanczyk addresses an area of pro- to pre-B-lymphocyte development that developmental biologists frequently neglect, namely the regulation of energy metabolism, specifically glycolysis and oxidative phosphorylation. The hypoxia-inducible transcription factor HIF1α plays an important role in early B-cell development by promoting glycolysis in B-cell progenitors. By contrast, Urbanczyk and colleagues have shown that the cell surface expression of the pre-B cell receptor down-regulates EFhd1, a Ca2^+^-binding protein that localises on the inner mitochondrial membrane and that limits glycolysis in pro-B cells. They therefore speculate on the importance of Ca2^+^ fluctuation-mediated mitochondrial flashes (mitoflashes) for the pro- to pre-B-cell transition [17].

## Concluding Remarks

**Figure 2:**
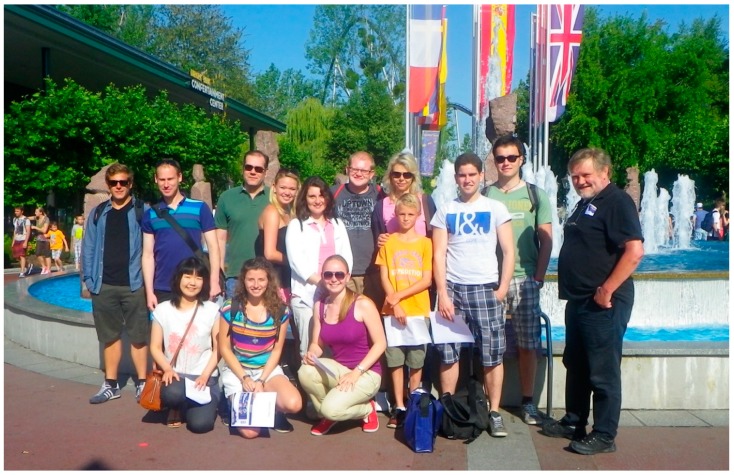
Ton stands to one side and lets the students take centre stage.

Many articles in this Special Issue concern the research interests of Ton. We take this opportunity to thank all of the DECIDE and non-DECIDE authors for their efforts in preparing these articles. Ton often commented on whether there was really a need to make a clear distinction between HSCs and their immediate progeny. HSCs can make an immediate choice of cell lineage, and as we might view HSCs as, at the very least, lineage affiliated, the distinction between HSCs and their immediate progeny becomes blurred. Ton and others showed that bone marrow stromal cells play a vital role in B-lymphocyte development. Appropriately, this issue highlights the increased interest and importance of bone marrow niches and hematopoietic cytokines in instructing lineage affiliation and cell differentiation, rather than HSCs and progenitors following a wired/prescribed developmental pathways in a stochastic manner. Some hematopoietic cytokines, including the ligand for FMS-like tyrosine kinase 3 (FL, myeloid versus lymphoid, as shown by Ton and colleagues), erythropoietin (erythroid), macrophage colony stimulating factor (CSF; monocyte), and granulocyte-colony stimulating factor (granulocyte) can instruct the HSC fate. There has been a long debate about whether IL-7 is instructive for B lymphocyte development. One of Ton’s last papers addressed this matter by showing that IL-7 and FL enhance the production of already “pre-decided” (committed) B-cell progenitors. IL-7 promoted their survival, whereas FL made these progenitors proliferate [18]. Opinions about IL-7 and B-cell niches and metabolism are also very important for our understanding of normal immunity, and possibly autoimmunity. For Ton, science was always meant to be fun; he did this by inspiring and encouraging his colleagues and students. This is perhaps best exemplified by a group photograph of Ton’s lab, taken in 2014 (Figure 2).

Finally, with new research tools becoming available, in particular single cell transcriptomic and proteomic analysis, it does seem that we are moving towards an entirely new architecture for haematopoiesis. This will guarantee that there will be lots of fun in the future trying to dissect this fascinating cell lineage. As emphasized in the review by Sánchez-García, a new view on normal haematopoiesis also has important implications for the way we view the initiation of cancer, perhaps as a fixed lineage-choice in a LSC/cancer stem cell that an oncogenic event wires. When considered in the context of this view of cancer initiation, understanding how to “normalise” the behaviour of LSCs and other cancer stem cells might give rise to potentially valuable therapeutic leads.

## Figures and Tables

**Figure 1:**
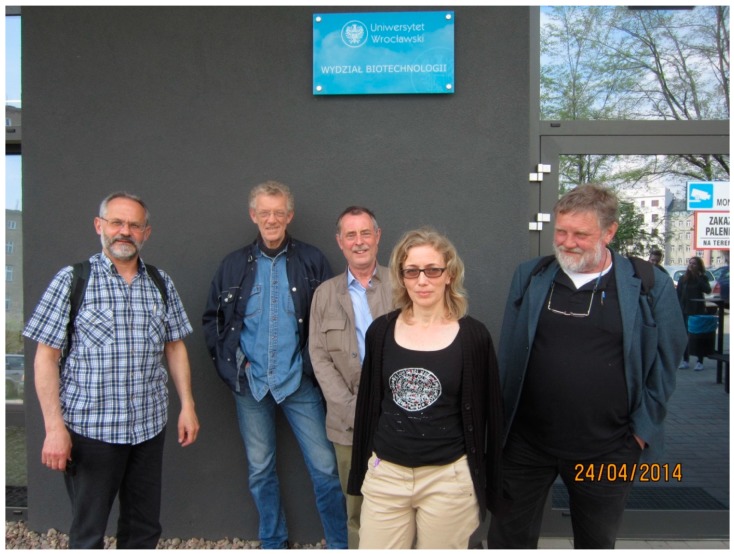
(L–R) Andrew Kutner, Rhodri Ceredig, Geoffrey Brown, Eva Marcinkowska, and Antonius (Ton) Gerardus Rolink at the Wroclaw DECIDE (decision-making within cells and differentiation entity therapies) consortium meeting in April 2014.
